# A centrosome-localized calcium signal is essential for mammalian cell mitosis

**DOI:** 10.1096/fj.201901662R

**Published:** 2019-11-02

**Authors:** Nordine Helassa, Charlotte Nugues, Dayani Rajamanoharan, Robert D. Burgoyne, Lee P. Haynes

**Affiliations:** Department of Cellular and Molecular Physiology, Institute of Translational Medicine, University of Liverpool, Liverpool, United Kingdom

**Keywords:** GCaMP, regulation, cell division

## Abstract

Mitosis defects can lead to premature ageing and cancer. Understanding mitosis regulation therefore has important implications for human disease. Early data suggested that calcium (Ca^2+^) signals could influence mitosis, but these have hitherto not been observed in mammalian cells. Here, we reveal a prolonged yet spatially restricted Ca^2+^ signal at the centrosomes of actively dividing cells. Local buffering of the centrosomal Ca^2+^ signals, by flash photolysis of the caged Ca^2+^ chelator diazo-2-acetoxymethyl ester, arrests mitosis. We also provide evidence that this Ca^2+^ signal emanates from the endoplasmic reticulum. In summary, we characterize a unique centrosomal Ca^2+^ signal as a functionally essential input into mitosis.—Helassa, N., Nugues, C., Rajamanoharan, D., Burgoyne, R. D., Haynes, L. P. A centrosome-localized calcium signal is essential for mammalian cell mitosis.

Mitosis is the mechanism whereby individual cells make duplicate copies of themselves, and it underpins the growth, development, and tissue repair of mammals (1). This is a fundamental cellular activity, defects in which can lead to premature ageing (2), aneuploidy, mitotic cell death, and cancer (3). Much is known regarding key regulatory systems that ensure fidelity during cell division with cyclin-based checkpoints (4, 5) and mitotic-specific kinases (6) making up the majority of the characterized regulatory network. Ca^2+^ is a universal intracellular second messenger that is found in all plants and animals (7). In most species it has a similarly ubiquitous role in controlling cellular behavior. Over 30 yr ago the role of Ca^2+^ during mitosis was investigated, initially using plant cells (8, 9) and model invertebrate systems amenable to micromanipulation and imaging techniques (10, 11). These studies observed both transient and global Ca^2+^ signals that appeared to correlate with specific mitotic events (nuclear envelope breakdown, metaphase-anaphase transition, cytokinesis entry). Furthermore, various functional experiments manipulating cytoplasmic Ca^2+^ during mitosis in these model systems strongly argued for an essential role for Ca^2+^ during normal mitotic progression (10, 12–14). When similar experiments were attempted in somatic mammalian cells the results were inconclusive (15–20) and, in particular, the presence of mitotic Ca^2+^ signals could not be confirmed despite the fact that Ca^2+^-binding proteins including calmodulin are required (21, 22).

In this study we have investigated the role of Ca^2+^ during mitosis in mammalian cells by developing a refined molecular toolkit to test whether hitherto undetected but highly localized Ca^2+^ signals could be important for mitosis. Our approach was to take proteins implicated in specific mitotic events and tag them with the monomeric GFP genetically encoded calcium indicator (GCaMP)6s Ca^2+^ sensor (23, 24). Our aim was to spatially restrict a high-affinity Ca^2+^ probe to mitosis-specific locations in dividing cells, which we hypothesized would permit the detection of mitosis-specific Ca^2+^ signals. Subsequent studies determined the functional significance of a centrosome-localized Ca^2+^ signal for mitosis.

## MATERIALS AND METHODS

### Plasmids

pTagRFP-actin was purchased from Evrogen (Moscow, Russia). pcDNA3.1(+) was purchased from Thermo Fisher Scientific (Waltham, MA, USA). pGP-CMV-GCaMP6s was a gift from Douglas Kim (plasmid 40753; Addgene, Watertown, MA, USA). pHIV-dTomato was a gift from Bryan Welm (plasmid 21374; Addgene). pMDLg/pRRE and pRSV-Rev were gifts from Didier Trono (plasmids 12251 and 12253; Addgene). pCMV-VSV-G was a gift from Bob Weinberg (plasmid 8454; Addgene). pANT7_cGST–centrosomal protein of 135 kDa (CEP135) was purchased from the DNASU Plasmid Repository (HsCD00640042; Biodesign Institute, Arizona State University, Tempe, AZ, USA) (25).

### Generation of pcDNA3.1(+)-actin-GCaMP6s and pmCherry-CEP135

To create pcDNA3.1(+)-actin-GCaMP6s, we first amplified the human cytoplasmic β-actin gene by PCR from pTagRFP-actin using New England Biolabs Phusion polymerase (forward, 5′-ATATAAGCTTACCATGGATGATGATATCGCCG-3′; reverse, 5′-ATGGATCCGAAGCATTTGCGGTGGA-3′) and cloned it into pcDNA3.1(+) by restriction ligation using *Hin*dIII-*Bam*HI and T4 DNA ligase (New England Biolabs, Ipswich, MA, USA). Then, the GCaMP6s gene was amplified by PCR from pGP-CMV-GCaMP6s using Phusion polymerase (forward, 5′-ATATGCGGCCGCATGACTGGTGGACAGCAAATG-3′; reverse, 5′-ATATGGGCCCTCACTTCGCTGTCATCATTTGTAC-3′) and cloned into pcDNA3.1(+)-actin by restriction ligation using *Not*I-*Apa*I and T4 DNA ligase. Integrity and localization of actin-GCaMP6s in mammalian cells was verified by Western blot and immunofluorescence (see below). To create pmCherry-CEP135, we first amplified the cep135 gene by PCR from pANT7_cGST-CEP135 using Phusion polymerase (forward, 5′-GGGCCCAATGACTACAGCTGTAGAGAG-3′; reverse, 5′-GGATCCCTACACATTTCTATGTTCAGGAG-3′) and cloned into pmCherry-C1 by restriction ligation using *Apa*I-*Bam*HI and T4 DNA ligase. All molecular constructs were verified by DNA sequencing (Sequencing Service, University of Dundee, United Kingdom).

### Cell culture and synchronization

Cells were cultured in DMEM [human cervical epithelial (HeLa) cells and human embryonic kidney 293T (HEK293T) cells] or DMEM-F12 [human neuroblastoma (SH-SY5Y) cells] containing 10% (v/v) heat-inactivated fetal bovine serum (Thermo Fisher Scientific), 1% (v/v) nonessential amino acids (Thermo Fisher Scientific), and penicillin and streptomycin (100 U/ml and 100 μg/ml, respectively) at 37°C in an atmosphere of 5% CO_2_. Cells were synchronized with 2 mM thymidine for 20–24 h, released with 25 μM deoxycytidine for 5–6 h (transfection with Lipofectamine 2000 was performed during this step when necessary, following the manufacturer’s recommendations) and blocked with either 2 mM thymidine (interphase block), 10 μM RO-3306 (G2/M block), or 35 ng/ml nocodazole (prometaphase block) for 15–24 h. Cells were washed 5 times with culture medium to release the block before confocal imaging.

### Validation of actin-GCaMP6s as an actin-targeted calcium sensor: immunofluorescence staining

Cells cotransfected with pcDNA3.1(+)-actin-GCaMP6s and pTagRFP-actin were fixed with 4% (w/v) formaldehyde (in PBS) for 15 min, washed with PBS, and permeablized with 1% (w/v) bovine serum albumin and 0.1% (v/v) Triton X-100 (in PBS) for 5 min. Cells were washed with PBS, blocked in 5% (w/v) bovine serum albumin for 10 min, and immunostained using mouse monoclonal anti–green fluorescent protein (GFP) antibody (Roche, Basel, Switzerland) followed by Alexa Fluor 647 phalloidin (Thermo Fisher Scientific) or anti-mouse Alexa Fluor 568 for 1 h. Cells were washed with PBS and treated with AlexaFluor 488 goat anti-mouse secondary antibody (Thermo Fisher Scientific) for 1 h. Cells were washed with PBS and coverslips were mounted on slides using Prolong Gold DAPI anti-fade glycerol (Thermo Fisher Scientific). Microscopic observation was made on a Zeiss LSM 800 (Carl Zeiss, Oberkochen, Germany) with Airyscan confocal microscope equipped with a Zeiss AxioObserver Z1, a ×63/1.4 Plan-Apochromat oil immersion objective, and diode lasers as excitation light source (405 nm for DAPI; 488 nm for actin-GCaMP6s; 561 nm for red fluorescent protein (RFP)–actin; 640 nm for phalloidin). Emitted light was collected through variable secondary dichroics onto a gallium arsenide phosphide–photomultiplier tube detector. Images were acquired using Zen Blue software (Carl Zeiss) and analyzed on ImageJ (National Institutes of Health, Bethesda, MD, USA) after Airyscan processing.

### Western blot analysis

HeLa cells transfected with GCaMP6s or actin-GCaMP6s were lysed using RIPA buffer. Proteins from the lysates were separated on SDS-PAGE [NuPAGE 4–12% (w/v) Bis-Tris, NuPAGE 3-(*N*-morpholino)propanesulfonic acid (MOPS) SDS running buffer; Thermo Fisher Scientific] and electrophoretically transferred onto Li-Cor Odyssey nitrocellulose membranes (Li-Cor Biosciences, Lincoln, NE, USA). Membranes were blocked with 5% (w/v) nonfat milk in Tris-buffered saline with Tween-20 (TBST) for 30 min and incubated with mouse monoclonal anti-GFP primary antibody (1:1000, mouse anti-GFP, 11814460001, clones 7.1 and 13.1, lot 10521400; Roche) for 1 h. Excess primary antibody was removed by TBST washes before incubation with peroxidase-conjugated anti-mouse IgG secondary antibody (1:1000; MilliporeSigma, Burlington, MA, USA) for 1 h at room temperature. Excess secondary antibody was removed by TBST washes, and antibody binding was detected using Pierce ECL Western blotting substrate (Thermo Fisher Scientific) on a ChemiDoc XRS+ (Bio-Rad, Hercules, CA, USA).

### Calcium imaging

HeLa cells stably expressing actin-GCaMP6s were plated on 35-mm glass-bottom dishes and challenged with 10 μM ionomycin or 500 μM histamine. Fluorescence over time was recorded on a 3i Marianas spinning-disk confocal microscope (3i, Denver, CO, USA) equipped with a Zeiss AxioObserver Z1, a ×40/1.3 Plan-Apochromat oil immersion objective, and a 3i Laserstack as excitation light source (488 nm for actin-GCaMP6s). Emitted light was collected through a single bandpass filter (CSU-X filter wheel; Yokogawa, Tokyo, Japan) onto a complementary metal-oxide semiconductor (CMOS) camera (Orca Flash 4.0; Hamamatsu, Hamamatsu, Japan). Images were collected at 1 frame every second (histamine) or every 5 s (ionomycin) using SlideBook v.6 software (3i) and processed on ImageJ. Data obtained from 10 cells was normalized and plotted on Prism 6 (GraphPad Software, La Jolla, CA, USA).

### Generation of stable HeLa cells expressing actin-GCaMP6s

Actin-GCaMP6s gene was amplified by PCR from pcDNA3.1(+)-actin-GCaMP6s using Phusion polymerase (forward, 5′-CATCATCTAGAGCTGGCTAGCGTTTAAAC-3′; reverse, 5′-GTAGTAACGTTCTGATCAGCGGGTTTAAAC-3′) and cloned into pHIV-dTomato by restriction ligation using *Xba*I with AclI or *Cla*I and T4 DNA ligase. During the process, dTomato is replaced by actin-GCaMP6s. All molecular constructs were verified by DNA sequencing (Sequencing Service). To generate stable HeLa cells expressing actin-GCaMP6s, we used a third-generation lentivirus system: pHIV-actin-GCaMP6s (20 μg) was cotransfected with pMDLg/pRRE (10 μg), pRSV-Rev (5 μg), and pCMV-VSV-G (6 μg) using Lipofectamine 2000 following the manufacturer’s recommendations in HEK293T cells (10-cm tissue culture dish). Supernatant containing the lentivirus particles was collected 48 h post-transfection, centrifuged, and clarified by syringe filtration (0.45 μm). HeLa cells were infected with actin-GCaMP6s lentivirus in a 6-well plate for 24 h. The stable cell line was then expanded and used for experiments.

### Localization of actin-GCaMP6s and centrosomes using live-cell confocal imaging

HeLa, SH-SY5Y, and HEK293T cells were plated on 35-mm glass-bottom culture dishes and synchronized using double thymidine (interphase) or thymidine-nocodazole (mitotic) blocks as described above. During the first release, cells were cotransfected with actin-GCaMP6s and mCherry-CEP135 using Lipofectamine 2000 following the manufacturer’s recommendations. Cells were imaged on a Zeiss LSM 800 laser scanning confocal microscope equipped with a Zeiss AxioObserver Z1, a ×63/1.4 Plan-Apochromat oil immersion objective, and diode lasers as excitation light source (488 nm for actin-GCaMP6s; 561 nm for mCherry-CEP135). Emitted light was collected through variable secondary dichroics onto a gallium arsenide phosphide–photomultiplier tube detector. Images were acquired using Zen Blue software and processed on ImageJ.

### Laser flash photolysis of diazo-2-acetoxymethyl ester (caged calcium chelator) in live-cell confocal imaging

HeLa cells stably expressing actin-GCaMP6s were plated on 35-mm glass-bottom dishes and synchronized using thymidine-nocodazole protocol. Before the second release, cells were loaded with 2 μM diazo-2-acetoxymethyl ester (diazo-2-AM; a kind gift from Alexei Tepikin, University of Liverpool) for at least 30 min at 37°C.

Cells were then released and examined on a 3i Marianas spinning-disk confocal microscope equipped with a Zeiss AxioObserver Z1, a ×40/1.3 Plan-Apochromat oil immersion objective, and a 3i Laserstack as excitation light source (405 nm for diazo-2-AM photolysis; 488 nm for actin-GCaMP6s). Emitted light was collected through single bandpass filters (CSU-X filter wheel; Yokogawa) onto a CMOS camera (Orca Flash 4.0; Hamamatsu). Experiments were carried out at 37°C and 5% (v/v) CO_2_ (incubation chamber from Okolab, Naples, Italy).

Photolysis of diazo-2-AM was performed by illumination with 405 nm laser light at 10% power for 10 ms for rapid chelation of Ca^2+^ at the centrosome or cytoplasm (elliptical region of interest) after 1–3 frames. Then, cell division was monitored by collecting images every minute using SlideBook v.6 software and processed on ImageJ and GraphPad Prism 6.

### Pharmacological treatments in live-cell confocal imaging

HeLa and SH-SY5Y cells were plated onto 24-well tissue culture plates and synchronized with double thymidine or thymdine–RO-3306 protocols as described above. After the second release, cells were treated with 2 μM thapsigargin, 25 nM concanomycin-A, 10 mM caffeine, 10 μM BTP2 (YM-58483), or vehicle controls. Cells were imaged on a 3i Marianas spinning-disk confocal microscope equipped with a Zeiss AxioObserver Z1 and a ×10/0.45 Plan-Apochromat objective. Experiments were carried out at 37°C and 5% (v/v) CO_2_ (incubation chamber from Okolab). Cell division was monitored by collecting transmitted light images onto a CMOS camera (Orca Flash 4.0; Hamamatsu) every 5 min using SlideBook v.6 software and processed on ImageJ.

### Transmission electron microscopy

Samples were prepared for transmission electron microscopy (TEM) as follows. HeLa cells were plated on a 10-cm dish and synchronized using the thymidine-nocodazole protocol as described above. Mitotic cells (shake-off method) were fixed in 2.5% (w/v) glutaraldehyde in 0.1 M phosphate buffer pH 7.4 (PB) in Pelco Biowave (Ted Pella, Redding, CA, USA) 1 min on, 1 min off, 1 min on, 100 W, 20 Hg. Cells were then washed twice in 0.1 M PB before embedding in 3% (w/v) agarose. Agarose embedded cell pellets were cut into small cubes before being postfixed and stained with reduced osmium [2% (w/v) osmium tetroxide in distilled H_2_O and 1.5% (w/v) potassium ferrocyanide in 0.1 M PB] in Biowave 20 s on, 20 s off, 20 s on, 20 s off, 20 s on, 100 W, 20 Hg. This was followed by a second osmication step [2% (w/v) in double-distilled H_2_O (ddH_2_O), 20 s on, 20 s off, 20 s on, 20 s off, 20 s on, 100 W, 20 Hg]. Samples were incubated overnight at 4°C in aqueous 1% (w/v) uranyl acetate. To prevent precipitation artifacts, the cells were washed for a minimum of 5 times for 3 min each with ddH_2_O between each of the staining steps described. The next day, cells were washed in ddH_2_O before dehydrated in a graded ethanol series of 30, 50, 70, 90% (v/v) in ddH_2_O for 5 min each, followed by 2 times 5 min 100% ethanol. Samples were then infiltrated with medium premix resin (TAAB Laboratories, Aldermaston, United Kingdom) at ratios of 1:1 with resin:100% ethanol for 30 min, and finally samples were incubated in 100% resin twice for 30 min, before embedding the pellets in fresh 100% resin in silicone molds and Beem capsules. Samples were cured for 48 h at 60°C.

Ultrathin serial section (70–75 nm) were cut on an UC6 ultramicrotome (Leica Microsystems, Buffalo Grove, IL, USA) and collected on formvar coated copper grids before viewing at 120 KV in a FEI Tecnai G^2^ Spirit (Thermo Fisher Scientific). Images were taken using a MegaView III camera (Emsis, Münster, Germany) using analysis software at various magnifications. Multiple image alignment was used on some images to create a high-resolution overview of areas of interest.

### Data analysis and statistics

Results are expressed as means ± sem unless indicated otherwise. All experiments were performed at least in triplicates. Number of experiments and total number of cells analyzed are given in the figure legends. Significance level was obtained using an unpaired, 2-tailed Student’s *t* test (GraphPad Prism 6 software).

## RESULTS AND DISCUSSION

**Figure 1:**
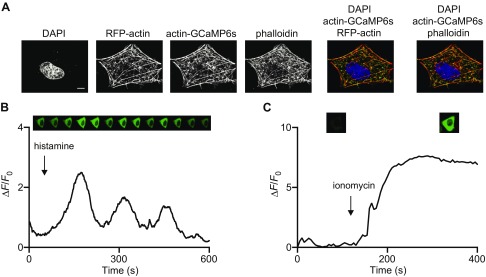
An actin-GCaMP6s Ca^2+^ sensor colocalizes with actin and responds to intracellular Ca^2+^ signals. *A*) Immunofluorescence confocal microscopy images of HeLa cells expressing RFP-actin and actin-GCaMP6s (DAPI, blue; RFP-actin or Alexa Fluor 647 phalloidin, red; actin-GCaMP6s, green). Scale bar, 10 μm. *B*, *C*) Representative traces of HeLa cells expressing actin-GCaMP6s stimulated with 500 μM histamine (*B*) or 10 μM ionomycin (*C*). Corresponding live-cell confocal images are presented in the top panel.

**Figure 2:**
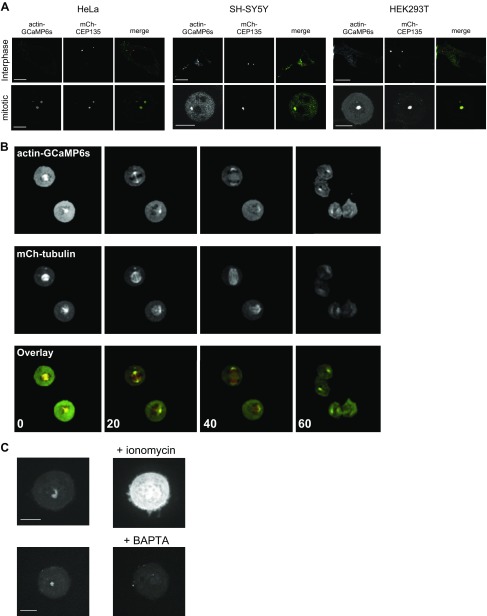
An actin-GCaMP6s probe detects a focal, persistent, and specific Ca^2+^ signal at the centrosomes of mammalian cells during mitosis. *A*) Representative confocal images of HeLa, SH-SY5Y, and HEK293T cells expressing actin-GCaMP6s (green) and mCherry-CEP135 (mCh-CEP135; red) in interphase and mitosis. Scale bar, 10 μm. *B*) Live-cell confocal images of HeLa cells expressing actin-GCaMP6s (green) and mCherry-tubulin (mCh-tubulin; red). Colocalization appears yellow in the overlay images. Time stamps are displayed in minutes in the overlay images only. *C*) Representative confocal images of HeLa cells expressing actin-GCaMP6s challenged with 10 μM ionomycin or 10 μM 1,2-*bis*(2-aminophenoxy)ethane-*N,N,N′,N′*-tetraacetic acid tetrakis (acetoxymethyl ester) (BAPTA-AM). Scale bars, 10 μm.

Actin has a well-documented role in forming the contractile ring necessary for constriction of the plasma membrane (PM) late in mitosis during telophase as the 2 nascent daughter cells prepare for physical separation (26). Initial validation of actin-GCaMP6s demonstrated that when expressed in interphase HeLa cells it colocalized extensively with cellular actin filaments (**Fig. 1*A***) and migrated at the predicted MW on SDS-PAGE (Supplemental Fig. S1*A*). In functional tests of actin-GCaMP6s’s ability to respond to Ca^2+^, we applied the Ca^2+^ mobilizing agonist histamine, which elicits well-documented Ca^2+^ oscillations in HeLa cells (Fig. 1*B* and Supplemental Fig. S1*B*). We also challenged transfected cells with ionomycin and Ca^2+^ to elevate cytoplasmic Ca^2+^ chronically (Fig. 1*C* and Supplemental Fig. S1*B*). In both instances we observed robust increases in actin-GCaMP6s fluorescence with histamine driving the expected oscillatory behavior. We also confirmed that actin-GCaMP6s expression had no deleterious effect on cell division (overall percentage of healthy cells completing mitosis and mean time taken to transit mitosis) when compared with control, untransfected cells (Fig. 1*C*). Remarkably, when expressed in HeLa cells, actin-GCaMP6s detected a highly localized Ca^2+^ signal during mitosis (**Fig. 2**). Studies in nonmitotic and mitotic cells have characterized the centrosome as having the ability to organize not only the microtubule network but also actin filaments (27–30). We therefore investigated the possibility that the probe was targeting to centrosomes. Using a centrosome-specific marker, mCherry-CEP135, we confirmed its colocalization with actin-GCaMP6s during mitosis (Fig. 2*A*). These data show both the presence of centrosome-associated actin and a region of elevated Ca^2+^ specifically at the centrosome in mitotic cells only (Fig. 2*A*). This phenomenon was observed in 3 cell types of widely differing lineages (HeLa, SH-SY5Y, and HEK293T), suggesting that it is universal. In these analyses there are examples in which an mCherry-CEP135–positive structure in a mitotic cell has no apparent corresponding actin-GCaMP6s signal (Fig. 2*A*: Hela cell during mitosis). This could be due to variation in local Ca^2+^ being experienced by individual centrosomes during mitosis such that some are highly fluorescent for actin-GCaMP6s, whereas others are less apparent. The Ca^2+^ detected by actin-GCaMP6s is visible from prophase and persists through to telophase (Fig. 2*B*). It therefore exhibits the unusual property of being spatially but not temporally focal. In order to prove that actin-GCaMP6s was registering a true Ca^2+^ signal at the centrosome, we tested the response of cells to elevation or depression of the cytoplasmic Ca^2+^ concentration (Fig. 2*C*). These experiments showed that actin-GCaMP6s was expressed extensively in cells (as would be expected for an actin-based construct) and that its fluorescence could be activated globally by ionomycin treatment in the presence of external Ca^2+^ (Fig. 2*C*). Similarly, the focal nature of the Ca^2+^ signal was confirmed by its disappearance when cells were incubated with the cell-permeant Ca^2+^ chelator, 1,2-*bis*(2-aminophenoxy)ethane-*N,N,N′,N′*-tetraacetic acid tetrakis (acetoxymethyl ester), which as a fast chelator would be effective at suppressing Ca^2+^ microdomains (Fig. 2*C*). Previous studies examining actin dynamics in mitotic cells (31, 32) did not observe actin specifically at centrosomes but highlighted that actin can be found in close proximity to spindle microtubules. We have shown that actin-GCaMP6s is expressed at many locations in mitotic cells (Fig. 2*C*) but detects a Ca^2+^ signal uniquely limited to the centrosome. Our observation of centrosomal actin during mitosis is new and consistent with that of centrosomal actin in interphase cells (27). We speculate that as well as nucleating actin in interphase cells, centrosomes may also exhibit this behavior during mitosis. We do not believe that the centrosome GCaMP signal is a nonspecific consequence of concentrating the probe into a restricted volume for various reasons. Firstly, GCaMP6s should have effectively undetectable fluorescence at resting Ca^2+^ levels (23). Secondly, we observe actin-GCaMP6s structures in mitotic cells, but they are not always present where mCherry-CEP135 is detected (Fig. 2*A* and discussion above). Thirdly, other studies using a PM-targeted GCaMP did not report fluorescence in the absence of a Ca^2+^ signal (33, 34). To provide further proof of this, we examined the fluorescence properties of a lysosomally targeted lysosome-associated membrane protein 1 (LAMP1)–GCaMP6s, based on a previous probe made in our laboratory (35). In live-cell analysis (Supplemental Fig. S2*A*), application of histamine generated Ca^2+^ oscillations in LAMP1-GCaMP6s–transfected cells (green region of interest) but not in untransfected control cells (red region of interest) or in a region of the culture dish devoid of cells (blue region of interest). The minimum fluorescence observed in transfected cells between calcium oscillations (resting cytoplasmic Ca^2+^) was only marginally higher that that observed for the control regions of interest. To further confirm the lack of detectable localized GCaMP6s fluorescence at resting cytosolic Ca^2+^ concentrations, HeLa cells were transfected with LAMP1-GCaMP6s and fixed for staining with an anti-GFP antibody (Supplemental Fig. S2*B*). The LAMP1 probe was targeted correctly and concentrated on the surface of lysosomes as expected (anti-GFP^568^ staining); however, no GCaMP6s fluorescence was detectable in these cells. Collectively these data show that, at resting cytoplasmic Ca^2+^ concentrations, GCaMP6s fluorescence is undetectable, even when the sensor is concentrated in a restricted volume such as on the surface of individual subcellular organelles (centrosomes, lysosomes, *etc.*).

**Figure 3:**
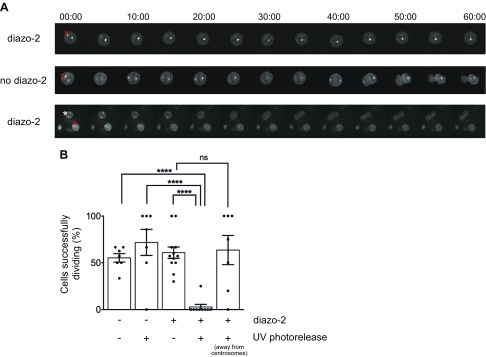
A centrosomal Ca^2+^ signal is essential for mitosis progression. *A*) Live-cell confocal images of HeLa cells stably expressing actin-GCaMP6s. Cells were UV-irradiated at the centrosomes (red star) or cytoplasm (white star) with or without preincubation with diazo-2-AM Ca^2+^ chelator. Time stamps are displayed in minutes:seconds. *B*) Quantitative analysis of the effect of Ca^2+^ depletion at the centrosomes on mitosis completion. Four different conditions were tested: HeLa cells stably expressing actin-GCaMP6s without UV irradiation (*n* = 39 cells and 7 independent experiments) and with UV irradiation at the centrosomes (*n* = 16 cells and 7 independent experiments, *P* < 0.0001); preincubated with diazo-2-AM without UV irradiation (*n* = 73 cells and 13 independent experiments, *P* < 0.0001) and UV irradiation at the centrosomes (*n* = 13 cells and 9 independent experiments, *P* < 0.0001) or in the cytoplasm (*n* = 21 cells and 7 independent experiments, *P* = 0.8479). Results are expressed as means ± sem; ns, not significant. ****P < 0.0001.

Having characterized a reproducible centrosomal Ca^2+^ signal, we next wanted to determine its functional significance. The focal nature of the Ca^2+^ meant that it would be impossible to manipulate using standard cell-permeant chelators without simultaneously interfering with all potential Ca^2+^-based signaling occurring in the cell. We therefore chose to utilize uncaging of the compound diazo-2-AM (36), a cell-permeant and UV photolysable Ca^2+^-chelator. This approach allowed us to activate the chelator only on 1 centrosome when cells were entering mitosis (**Fig. 3** and Supplemental Fig. S3 and Supplemental Movies S1 and S3). When diazo-2-AM–loaded cells were tracked following centrosomal UV irradiation, only 3% of healthy (viable, nondead) cells progressed normally through mitosis in comparison with 61% of nonirradiated diazo-2-AM–loaded viable control cells from the same experiments (Fig. 3). In control conditions with no diazo-2-AM loading and no UV irradiation, ∼55% of all viable cells progressed normally through mitosis. This reflects the fact that in chemically synchronized HeLa cell populations, a significant proportion of healthy cells fail to release from the chemical block and progress through mitosis. In these experiments, cells with no diazo-2 loading but in which the uncaging UV irradiation protocol was applied to actin-GCaMP6s–positive centrosomes, the number of healthy irradiated cells that progressed normally through mitosis was similar at 72% (Fig. 3 and Supplemental Movie S2). UV irradiation of the centrosome in the absence of diazo-2-AM therefore had no deleterious influence on mitosis progression. To exclude the possibility that diazo-2-AM activated over centrosomes was diffusing to another (noncentrosomal) cellular location and that buffering of Ca^2+^ here was responsible for inhibition of mitosis, we performed additional controls. In these experiments we loaded cells with diazo-2-AM and uncaged the chelator in a region of cytoplasm spatially distinct from the centrosomes (Fig. 3 and Supplemental Movie S3). When we photo-activated diazo-2-AM in noncentrosomal regions we observed no decrease in GCaMP6s fluorescence at centrosomes and no effect on normal progression through mitosis (64% of cells successfully dividing). In this particular example it was also possible to simultaneously irradiate and uncage diazo-2-AM over a centrosome of a second actin-GCaMP6s–expressing cell in the same field of view (Supplemental Movie S3) and to inhibit completion of mitosis. Collectively these data show that specific uncaging of a Ca^2+^ chelator over a single centrosome in a dividing mammalian cell is sufficient to suppress the centrosomal Ca^2+^ signal and precipitate an immediate block on further progression through mitosis. Furthermore, this effect is confined to the centrosome itself because activation of the chelator in a randomly selected area of cytoplasm did not affect mitosis progression. Another striking feature of these analyses is that the Ca^2+^ signal at centrosomes does not reappear following a relatively short (tens of milliseconds) UV uncaging protocol. Determining the precise nature of how centrosomal Ca^2+^ is generated and maintained throughout mitosis is beyond the scope of the present paper. It is nonetheless interesting to speculate about a possible mechanism. A recent study has shown that specific populations of immobile inositol 1,4,5-trisphosphate (IP_3_) receptors (IP_3_Rs) are responsible for active Ca^2+^ signaling at endoplasmic reticulum (ER)–PM membrane junctions (37). It has also been reported that ER Ca^2+^ release is regulated by actin polymerization (38) and that actin dynamics can influence IP_3_-mediated Ca^2+^ release during fertilization in starfish oocytes (39, 40). One hypothesis that we have is that centrosomal actin might perhaps be responsible for organizing IP_3_R signaling domains in close proximity to centrosomes in mitotic cells and that maintenance of these domains depends on continuity of the Ca^2+^ signal. Once the Ca^2+^ signal is disrupted, the connection between ER and centrosome is lost and cannot be reestablished. Future work will be directed at investigating such possibilities to provide a complete mechanistic explanation of centrosome Ca^2+^.

**Figure 4:**
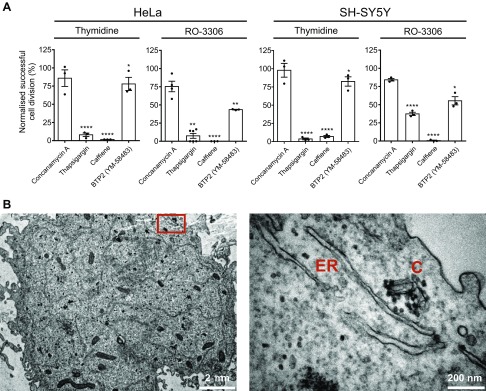
Ca^2+^ from the ER is required for mitosis progression. *A*) Effect of pharmacological treatments targeting Ca^2+^ signaling pathways on cell division in HeLa and SH-SY5Y cells synchronized at interphase (thymidine) or G2/M (RO-3306). All treatment data have been normalized to cell division observed in the appropriate vehicle control conditions (100%). Results are expressed as means ± sem. *P < 0.05, **P < 0.01, ****P < 0.0001. *B*) Representative TEM image of a mitotic HeLa cell at 6K or 60K magnification. C, centrosome.

Furthermore, we wanted to gather evidence for a likely source of Ca^2+^ that was feeding the centrosomal signal in mitotic cells. For this analysis we employed a series of standard and widely used pharmacological inhibitors of various Ca^2+^-mobilizing pathways (**Fig. 4*A*** and Supplemental Fig. S4). Many of these agents will have pleiotropic effects on cells, and they cannot be used to infer a direct influence on centrosomal Ca^2+^. We therefore restrict our interpretation of the data to provide circumstantial evidence as to a likely source of cellular or extracellular Ca^2+^ that is able to influence mitosis, which could be consistent with our observations of centrosomal Ca^2+^. Two independent methods of antagonizing IP_3_R-dependent ER Ca^2+^ release both significantly impeded completion of mitosis. Treatment of cells with the IP_3_R inhibitor caffeine (41) or the sarcoplasmic reticulum Ca^2+^-ATPase pump inhibitor thapsigargin (42) elicited significant impairment of mitosis completion (Fig. 4*A* and Supplemental Fig. S4). Caffeine at millimolar concentrations has been shown to specifically inhibit IP_3_R1 (41), consistent with this being the major isoform expressed in HeLa cells (43). Treatment of cells with the store-operated Ca^2+^ entry inhibitor, BTP2 (YM-58483) (44), elicited a small, if significant, increase in cells failing to progress through mitosis. The limited effect of BTP2 is perhaps not unexpected because store-operated Ca^2+^ entry through Orai channels is known to be down-regulated during mitosis (45). The lysosomal V-ATPase inhibitor concanamycin-A, which induces lysosomal Ca^2+^ depletion, was without effect on mitosis. Lysosomes have recently been characterized as important Ca^2+^-signaling platforms (46), although our data argue against a role for lysosomal Ca^2+^ release during mitosis. We employed 2 cell synchronization protocols in this part of the study, double thymidine (cells arrested in interphase) or thymidine–RO-3306 (cyclin-dependent kinase 1 inhibitor that arrests cells at the G2/M boundary). The data sets are complimentary for each protocol and indicate that the Ca^2+^ signal important for mitosis therefore occurs at some point during or following prophase. These experiments were additionally performed in parallel on 2 independent cell lines of divergent lineage, HeLa (human cervical epithelial cells) and SH-SY5Y (human neuroblastoma cells). The data sets for each cell line follow identical trends, indicating that ER Ca^2+^ is a universal requirement for mitosis progression in mammalian cells.

Finally, we examined the localization of centrosomes and ER in mitotic HeLa cells by TEM (Fig. 4*B* and Supplemental Fig. S4*B*) to look for evidence that these 2 cellular entities might exist in close proximity, consistent with a model in which the ER is the organelle driving centrosomal Ca^2+^. In plant cells (47) and invertebrate model organisms (48) the ER has been shown to associate closely with the mitotic apparatus and spindle poles, and in our studies we observed strands of ER closely apposed with centrosomes. The limited number of high-quality images of mitotic cells that we were able to generate for this analysis restricted us to a purely qualitative examination of the TEM data. This did however reveal that ER was the most frequently observed, morphologically distinguishable, cellular organelle located close to centrosomes, consistent with the pharmacological treatment data and our assertions regarding the source of centrosomal Ca^2+^.

## CONCLUSIONS

In this study we have employed spatial restriction of a genetically encoded Ca^2+^ sensor to detect a focally restricted centrosome-based Ca^2+^ signal in mitotic cells. Given that the Ca^2+^ binding proteins calmodulin (22) and centrin-2 (49) are required for mitosis, this work now provides the missing link that connects the cell biology of Ca^2+^ signaling with mitosis in mammalian cells and has important implications for furthering our understanding of normal growth, development, and ageing. Because centrins-2 and -3 are centrosome-associated Ca^2+^ binding proteins (50), this could be a clue as to why a centrosome-localized Ca^2+^ signal is of crucial importance. Centrosomally localized Ca^2+^ signaling is likely a universal phenomenon essential during mitosis and represents an entirely novel control mechanism that opens new avenues of investigation into modulating cell division.

## Supplementary Material

This article includes supplemental data. Please visit *http://www.fasebj.org* to obtain this information.

## Abbreviations

CEP135: centrosomal protein of 135 kDa
CMOS: complementary metal-oxide semiconductor
ddH_2_O: double-distilled H_2_O
diazo-2-AM: diazo-2-acetoxymethyl ester
ER: endoplasmic reticulum
GCaMP: monomeric GFP genetically encoded calcium indicator
GFP: green fluorescent protein
HEK293T: human embryonic kidney 293T
IP_3_: 1,4,5-trisphosphate
IP_3_R: IP_3_ receptor
LAMP1: lysosome-associated membrane protein 1
PB: phosphate buffer pH 7.4
PM: plasma membrane
RFP: red fluorescent protein
TBST: Tris-buffered saline with Tween-20
TEM: transmission electron microscopy
