# Supplementary material for: A centrosome-localized calcium signal is essential for mammalian cell mitosis
Source: FASEB J. 2019 Nov 2;33(12):14602–10. doi: 10.1096/fj.201901662R (PMC6910830; doi:10.1096/fj.201901662R)
Supplement: Supplementary file 1 [file fj.201901662R.sf1.pdf]

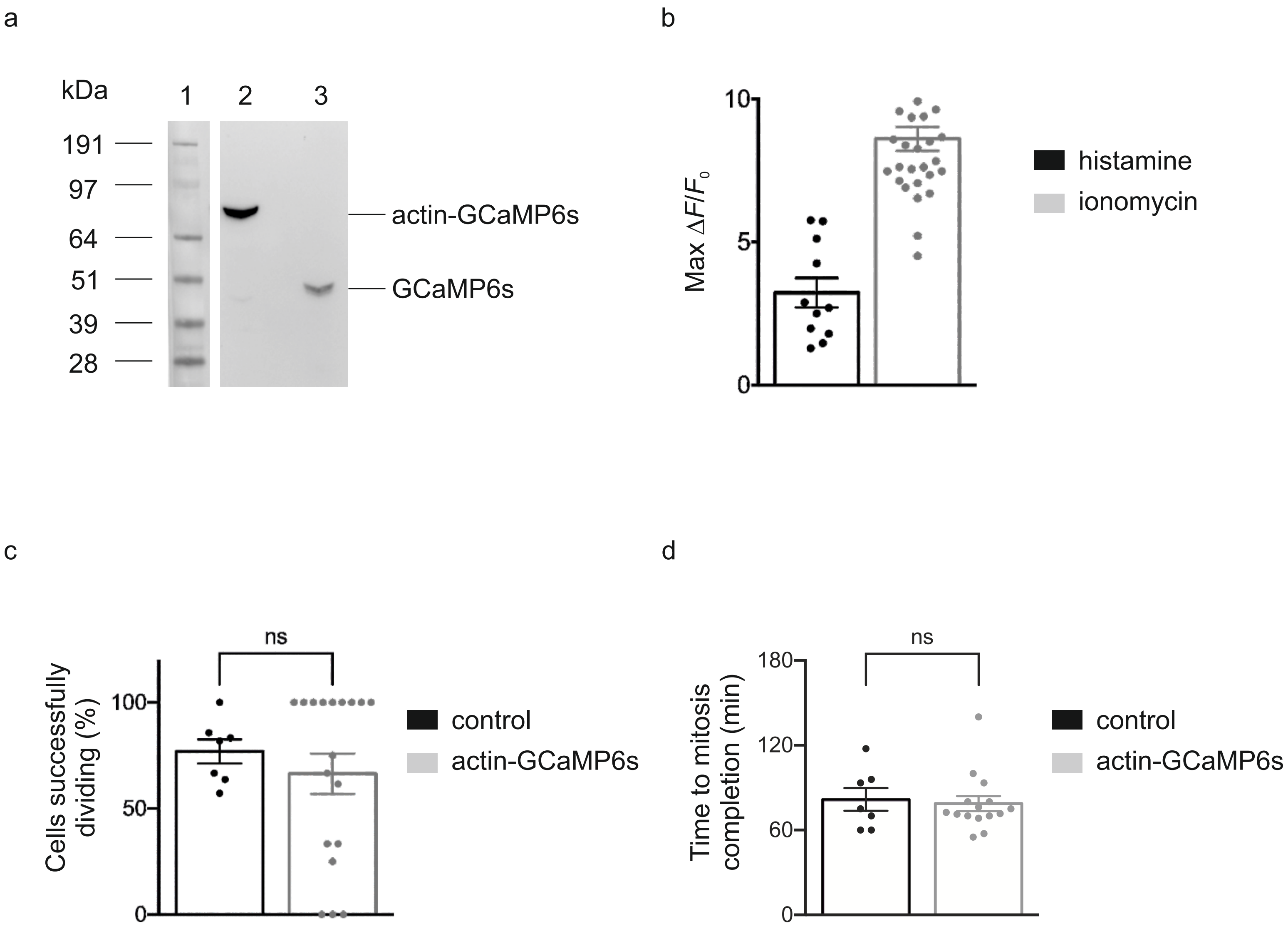

Figure S1. Characterisation of actin-GCaMP6s expression, functionality and cytotoxicity in HeLa cells. (*A*) HeLa cells expressing actin-GCaMP6s or GCAMP6s alone were processed for analysis by SDS-PAGE and Western blotting with anti-GFP antibody. Actin-GCaMP6s migrated at the predicted molecular weight of 91 kDa. (*B*) Peak actin-GCaMP6s fluorescence signal observed in cells treated with 500 μM histamine (n=11, N=3) or 10 μM ionomycin in the presence of 1.8 mM external Ca^{2+} (n=30, N=4). (*C*) HeLa cells expressing actin-GCaMP6s were synchronized with thymidine-nocodazole and released from cell cycle arrest. The number of cells successfully completing mitosis (defined as all healthy cells that entered mitosis and successfully completed cytokinesis) were scored as a % of the total number of healthy cells analysed (n=60, N=18) and compared to control non-transfected cells (n=46, N=7, p=0.5107). In all analyses there was a proportion of visible cell death likely due to the chemical block treatment. Non-viable cells (ignored from all cell counts) were identified as cells that failed to enter/complete mitosis (see above definition of healthy cells) in combination with having one or both of the following morphological features in brightfield images: cellular condensation, membrane rupture. (*D*) Data from (C) was additionally analysed to determine mitosis transit time (time from entry into Prophase to exit from cytokinesis) in control versus actin-GCaMP6s expressing cells. Delays in mitosis completion are often indicative of compromised cell viability. We observed no statistically significant difference in the average time taken for control or actin-GCaMP6s expressing cells to enter mitosis and exit cytokinesis. n is the total number of cells analysed, N the number of independent experiments performed and p the calculated probability (p-value). Results are expressed as mean ± SEM.
