# Supplementary material for: A centrosome-localized calcium signal is essential for mammalian cell mitosis
Source: FASEB J. 2019 Nov 2;33(12):14602–10. doi: 10.1096/fj.201901662R (PMC6910830; doi:10.1096/fj.201901662R)
Supplement: Supplementary file 2 [file fj.201901662R.sf2.pdf]

a

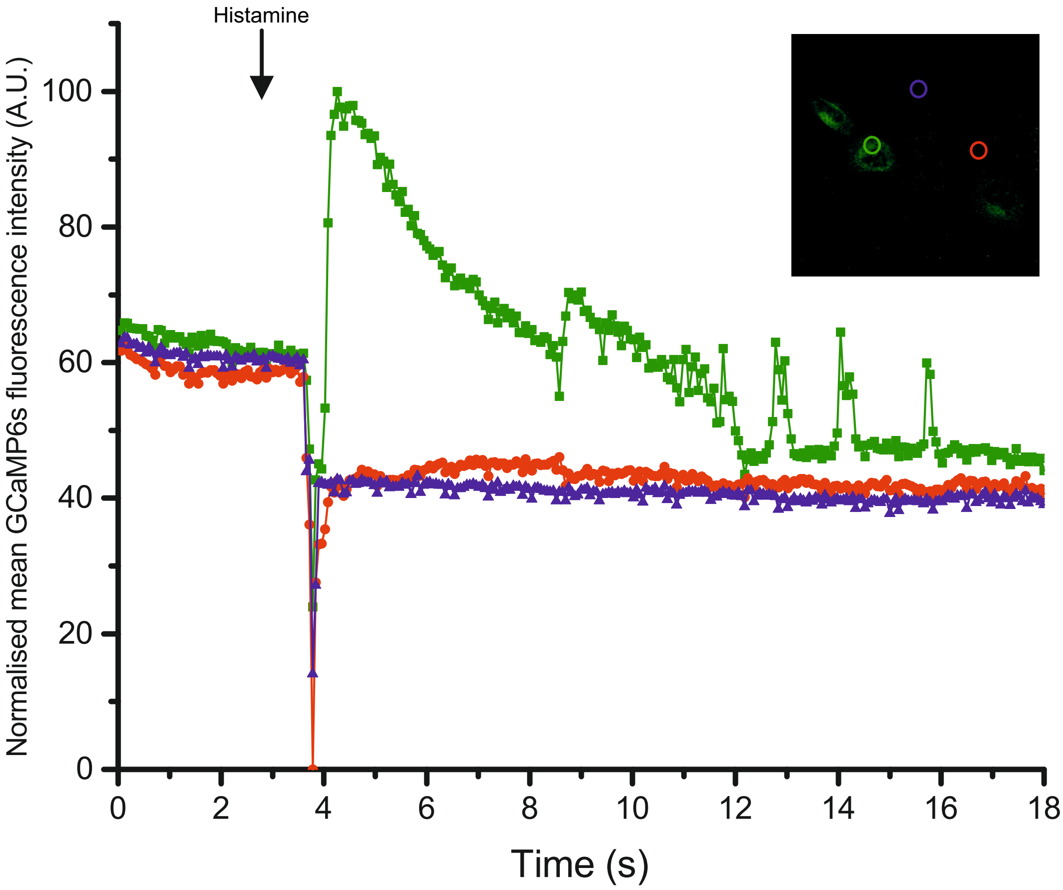

b

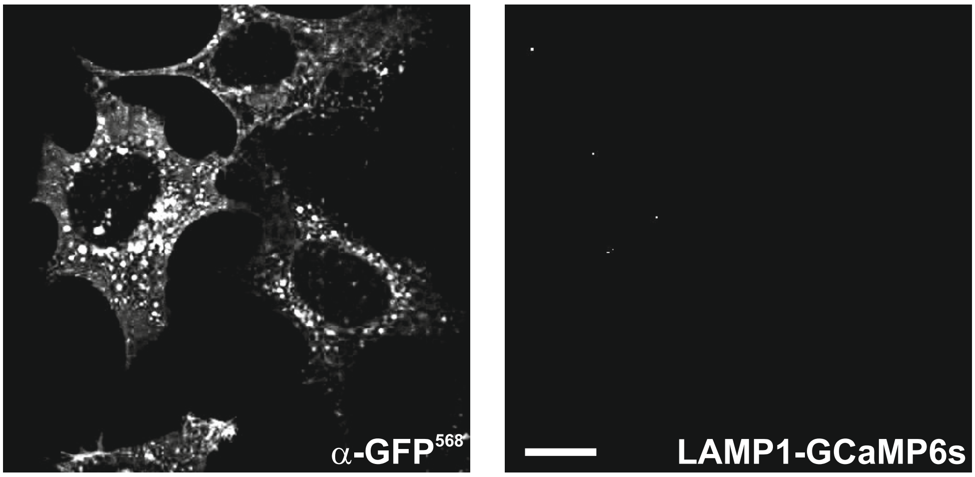

Figure S2. Targeting and concentration of GCaMP6s to a sub-cellular organelle does not produce detectable GCaMP fluorescence in the absence of elevated cytosolic Ca^{2+}. (*A*) HeLa cells were transfected with a GCaMP6s tagged variant of lysosomal membrane protein-1 (LAMP1-GCaMP6s). Live cells were imaged at a frame rate of 1 frame/60ms and 100μM Histamine applied at the time point indicated by the arrow. LAMP1-GCaMP6s fluorescence was determined in three regions of interest: Red circle – Cytoplasm of an untransfected control cell; Blue circle – An area of the culture dish devoid of cells; Green circle – A LAMP1-GCaMP6s expressing cell. (*B*) HeLa cells expressing LAMP1-GCaMP6s were fixed and stained with anti-GFP antibody followed by detection with and Alexa-568 conjugated secondary antibody. Cells were imaged with 488nm (GCaMP fluorescence excitation) and 561nm (Alexa fluorescence excitation) laser lines. Scale bar = 10μm.
