# Supplementary material for: A centrosome-localized calcium signal is essential for mammalian cell mitosis
Source: FASEB J. 2019 Nov 2;33(12):14602–10. doi: 10.1096/fj.201901662R (PMC6910830; doi:10.1096/fj.201901662R)
Supplement: Supplementary file 3 [file fj.201901662R.sf3.pdf]

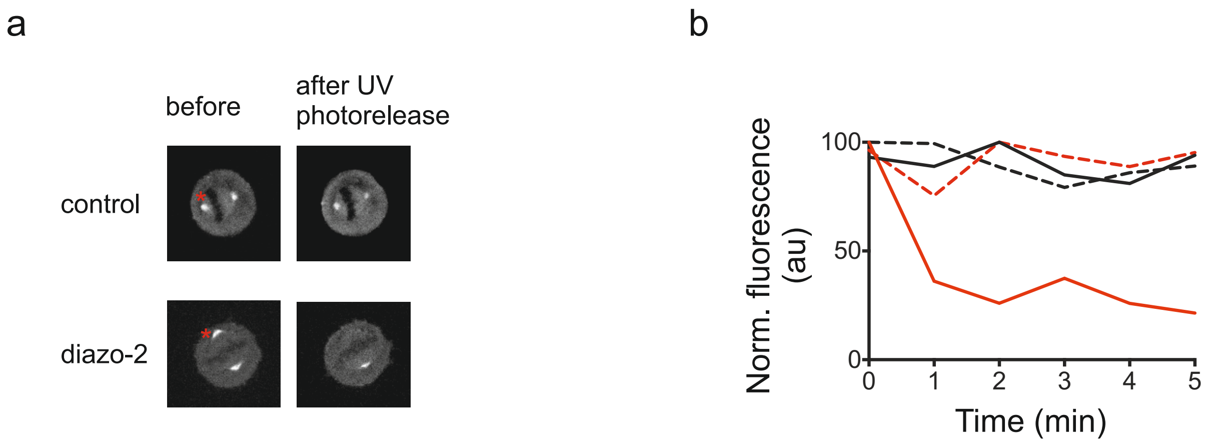

Figure S3. Flash photolysis of centrosomes during mitosis leads to a specific depletion of actin-GCaMP fluorescence only in diazo-2 loaded cells. (*A*) HeLa cells stably expressing actin-GCaMP6s were synchronized with thymidine-nocodazole and incubated with diazo-2 or left untreated (control). Cells were imaged at metaphase and a single centrosome UV irradiated (red asterisk). (*B*) Actin-GCaMP fluorescence traces corresponding to cells from (*A*). Fluorescence signal following UV irradiation (time = 0) at the irradiated (solid black line) and non-irradiated (dashed black line) centrosomes of the control cell from (*A*). Fluorescence signal at the irradiated (solid red line) and non-irradiated (dashed red line) centrosomes of the diazo-2 loaded cell from (*A*).
