# Supplementary material for: A centrosome-localized calcium signal is essential for mammalian cell mitosis
Source: FASEB J. 2019 Nov 2;33(12):14602–10. doi: 10.1096/fj.201901662R (PMC6910830; doi:10.1096/fj.201901662R)
Supplement: Supplementary file 4 [file fj.201901662R.sf4.pdf]

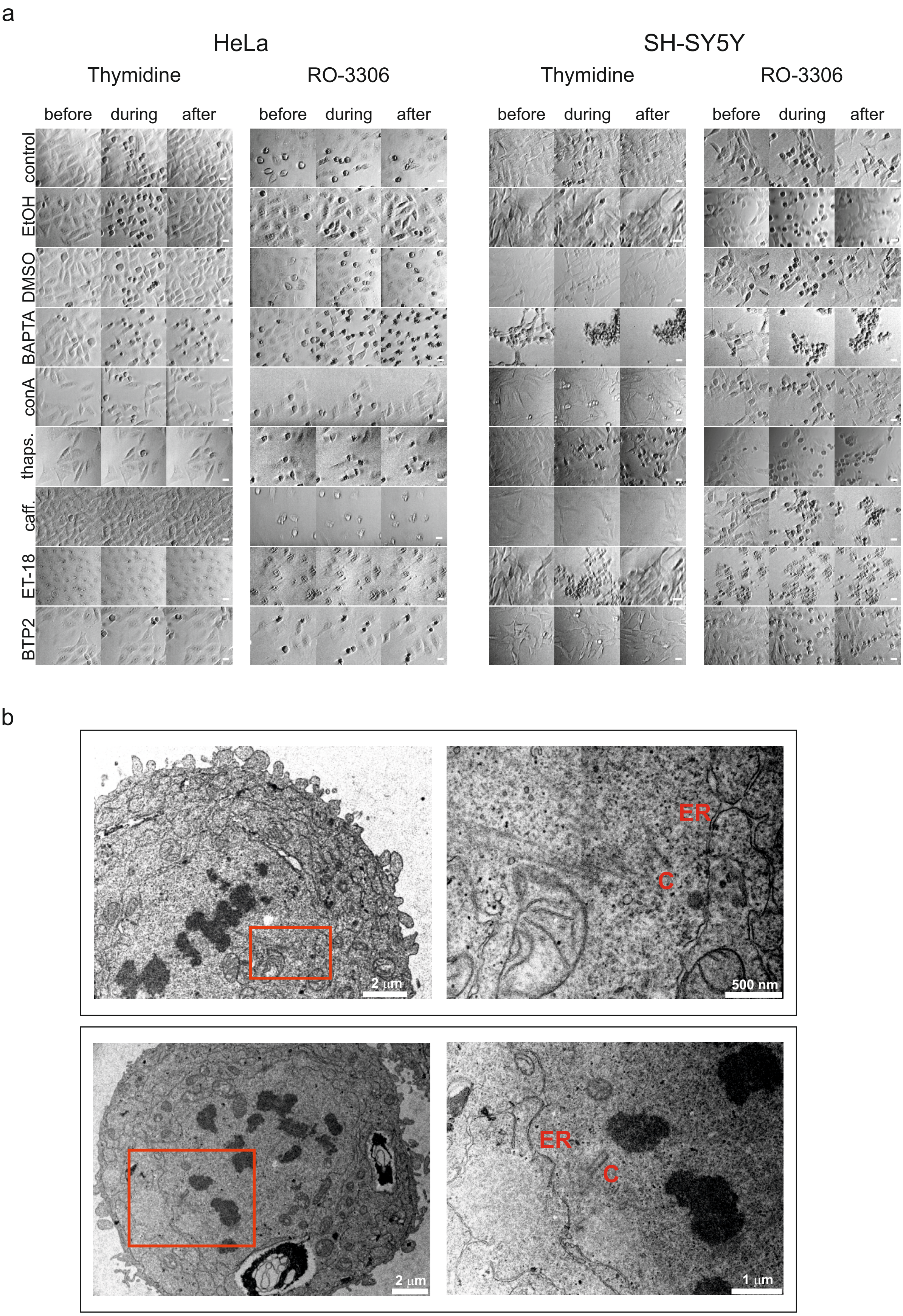

Figure S4. The endoplasmic reticulum is observed in close proximity to centrosomes in mitotic HeLa cells and pharmacological agents blocking ER Ca^{2+} signals induce mitotic arrest. (*A*) Brightfield images (before, during and after mitosis) of HeLa and SH-SY5Y cells synchronized with double thymidine or thymidine/RO-3306 and treated with various agents intended to disrupt specific cellular Ca^{2+} signaling pathways or vehicle controls (Ethanol (EtOH) and Dimethylsulfoxide (DMSO)). Caff., Caffiene; Thaps., Thapsigargin; ConA, Concanamycin A. The cell division results depicted in Fig. 4*A* histograms were derived from these data. (*B*) Examples of transmission electron micrographs taken from sections of mitotic HeLa cells. Endoplasmic reticulum ("ER") was often observed in close proximity to centrosomes ("C") in these samples. The higher magnification images shown in the right hand panel correspond to the regions of interest defined by red rectangles shown in the lower magnification pictures of the left hand panel.
