# Supplementary material for: A centrosome-localized calcium signal is essential for mammalian cell mitosis
Source: FASEB J. 2019 Nov 2;33(12):14602–10. doi: 10.1096/fj.201901662R (PMC6910830; doi:10.1096/fj.201901662R)
Supplement: Supplementary file 5 [file fj.201901662R.sd1.pdf]

Movie S1. Flash photolysis of Diazo-2 at centrosomes blocks mitosis. HeLa cells stably expressing actin-GCaMP6s were synchronized using thymidine-nocodazole and incubated with diazo-2. Cells were imaged at metaphase and a single centrosome UV irradiated (red asterisk).

Movie S2. UV-irradiation at centrosomes does not affect cell division. HeLa cells stably expressing actin-GCaMP6s were synchronized using thymidine-nocodazole. Cells were imaged at metaphase and a single centrosome UV irradiated (red asterisk).

Movie S3. Flash photolysis of Diazo-2 in a non-centrosomal region of cytoplasm does not block mitosis. HeLa cells stably expressing actin-GCaMP6s were synchronized using thymidine-nocodazole and incubated with diazo-2. Cells were imaged at metaphase and in one cell, a region of interest chosen in the cytoplasm at the edge of the metaphase plate (white asterisk). This region was UV irradiated. In the same field of view Diazo-2 was activated over a single centrosome (as for Movie S1) in a second actin-GCaMP6s expressing cell (red asterisk).
